# Time-Dependent ECG-AI Prediction of Fatal Coronary Heart Disease: A Retrospective Study

**DOI:** 10.3390/jcdd11120395

**Published:** 2024-12-08

**Authors:** Liam Butler, Alexander Ivanov, Turgay Celik, Ibrahim Karabayir, Lokesh Chinthala, Mohammad S. Tootooni, Byron C. Jaeger, Luke T. Patterson, Adam J. Doerr, David D. McManus, Robert L. Davis, David Herrington, Oguz Akbilgic

**Affiliations:** 1 Cardiovascular Section, Department of Internal Medicine, Wake Forest University School of Medicine, Winston-Salem, NC 27101, USA; 2 Center for Biomedical Informatics, University of Tennessee Health Sciences Center, Memphis, TN 38163, USA; 3 Health Informatics and Data Science, Loyola University Chicago, Maywood, IL 60660, USA; 4 Division of Public Health Science, Department of Biostatistics and Data Science, Wake Forest University School of Medicine, Winston-Salem, NC 27101, USA; 5 Department of Medicine, University of Massachusetts Chan Medical School, Worcester, MA 01655, USA

**Keywords:** fatal coronary heart disease, FCHD, ECG-AI, time-dependent risk, risk prediction, ECG-AI-Cox

## Abstract

**Background**: Fatal coronary heart disease (FCHD) affects ~650,000 people yearly in the US. Electrocardiographic artificial intelligence (ECG-AI) models can predict adverse coronary events, yet their application to FCHD is understudied. **Objectives**: The study aimed to develop ECG-AI models predicting FCHD risk from ECGs. **Methods (Retrospective)**: Data from 10 s 12-lead ECGs and demographic/clinical data from University of Tennessee Health Science Center (UTHSC) were used for model development. Of this dataset, 80% was used for training and 20% as holdout. Data from Atrium Health Wake Forest Baptist (AHWFB) were used for external validation. We developed two separate convolutional neural network models using 12-lead and Lead I ECGs as inputs, and time-dependent Cox proportional hazard models using demographic/clinical data with ECG-AI outputs. Correlation of the predictions from the 12- and 1-lead ECG-AI models was assessed. **Results**: The UTHSC cohort included data from 50,132 patients with a mean age (SD) of 62.50 (14.80) years, of whom 53.4% were males and 48.5% African American. The AHWFB cohort included data from 2305 patients with a mean age (SD) of 63.04 (16.89) years, of whom 51.0% were males and 18.8% African American. The 12-lead and Lead I ECG-AI models resulted in validation AUCs of 0.84 and 0.85, respectively. The best overall model was the Cox model using simple demographics with Lead I ECG-AI output (D1-ECG-AI-Cox), with the following results: AUC = 0.87 (0.85–0.89), accuracy = 83%, sensitivity = 69%, specificity = 89%, negative predicted value (NPV) = 92% and positive predicted value (PPV) = 55% on the AHWFB validation cohort. For this, the 2-year FCHD risk prediction accuracy was AUC = 0.91 (0.90–0.92). The 12-lead versus Lead I ECG FCHD risk prediction showed strong correlation (R = 0.74). **Conclusions**: The 2-year FCHD risk can be predicted with high accuracy from single-lead ECGs, further improving when combined with demographic information.

## 1. Introduction

Fatal coronary heart disease (FCHD) affects more than 650,000 people in the USA and is associated with more than 7 million deaths globally [1,2,3]. FCHD has shown increasing prevalence in adults aged 30–40 years [1,4]. FCHD often leads to sudden cardiac failure and is attributed to a history of cardiovascular disease, history of nonfatal myocardial infarction or underlying coronary disease risk factors [5,6,7]. Researchers have suggested that FCHD is highly correlated with sudden cardiac death (SCD) [7,8,9].

There have been developments in potential primary prevention when the risk of FCHD or SCD has been detected, often relying on the prevention or treatment of ischemic events, heart failure (HF), coronary artery disease (CAD), hypertrophic cardiomyopathy (HCM) or arrhythmias (including fatal arrhythmias). In most cases, the attributable prevention strategy is the use of implantable cardioverter defibrillators; however, there are cases where the underlying cardiovascular irregularity is clinically addressed via appropriate pharmacological therapies such as betablockers for long QT syndrome [10] and non-ischemic evaluation for those with anomalous coronary arteries [11,12]. However, early detection and prediction of the event remain challenging since risk modification strategy requires warning [13,14] to help reduce the chance of the event using an appropriate intervention strategy.

Artificial intelligence (AI) methods have become assets in detecting and predicting risk for different cardiovascular diseases due to the large availability of clinical data [15,16]. There is potential for the development of AI models for pre-screening during any clinical service to assess the risk of FCHD at an early enough stage to allow for prevention or timely therapeutics. Current research has tried to predict FCHD risk using clinical risk factors [17], ECG features as biomarkers [5,18] and cardiac imaging [19,20]. These, however, have limitations attributed to prediction performance, small sample sizes or require additional resources, which might not be accessible to everyone.

Prior studies have shown that ECG features (e.g., QRS amplitude, QRS duration, etc.) relate to risk for both FCHD and SCD [21,22,23]. In a study by Aro et al. [24], it was shown that the ECG-based risk score, based on six ECG risk markers, is independently associated with SCD and indeed improved the accuracy of identifying people at high risk of SCD. This risk score incorporated information from multiple ECG parameters. This showcases the importance of ECGs in detecting and, further, predicting the risk of fatal diseases. Raw ECG data may, therefore, be useful to predict sudden cardiac cessation since these are routinely collected in any clinical setting and are low cost and non-invasive [5]. In comparison to the study by Aro et al. [24], the use of raw ECGs reduces the requirement of ECG parameters being extracted and can help simplify model utilization while reducing data requirements. Simple raw 12-lead ECGs, when incorporated within deep learning frameworks, have been shown to be able to predict multiple cardiovascular diseases, including cardiomyopathies and heart failure (HF) [25,26,27,28]. Furthermore, recent research by our team and others has also successfully shown that such (and related) diseases can be predicted or detected at high accuracy using only Lead I from a 12-lead ECG [29,30,31,32,33]. The increase in ECG capabilities in smart devices (e.g., smart watches or fitness trackers) increases the utility for easier and less expensive testing or monitoring of risk, especially when there is a familial history of sudden death, in the younger generation or athletes. In addition, the use of single-lead based ECG-AI can also increase the remote monitoring of patients who are deemed to be at high risk (e.g., people with hypertrophic cardiomyopathy or left ventricular hypertrophy).

The goal of this research was to develop ECG-AI-based models for FCHD risk prediction for the general population that can help clinicians to monitor and assess risk for timely intervention. The specific aims of this research are to (i) develop and externally validate deep learning models that can predict risk for FCHD using both 12-lead and single-lead ECGs, (ii) assess the concordance of 12-lead vs. single-lead-ECG only models in FCHD risk prediction and (iii) perform time-dependent analysis of FCHD risk to identify time-points when our model is accurate and also allow for timely intervention (e.g., 2–5 year intervals).

## 2. Materials and Methods

### 2.1. Data Sources

This study used retrospective standard 12-lead 10 s time-voltage supine ECG data, demographic and clinical data, including co-morbidities (risk factors), for patients, which were obtained from the electronic health records (EHRs) at the University of Tennessee Health Science Center/Medical Center in Memphis, Tennessee (UTHSC) and at the Atrium Health Wake Forest Baptist (AHWFB), Winston-Salem, NC. The study was approved by the IRBs of both UTHSC and AHWFB.

### 2.2. Outcome Definition

Fatal coronary heart disease (FCHD) was defined as sudden cardiovascular failure. More specifically, in the case of FCHD, cardiovascular failure often refers to sudden cardiac activity cessation when the person was otherwise healthy before the event. In addition, the only medical evidence to support the cessation is a history of cardiovascular disease or additional risk factors that are often associated with coronary disease [5,6]. FCHD events were derived from a combination of ICD-9 codes 410, 427.5, 799 and ICD-10 codes I46, I46.2, I46.9, I21, and I25.x [5,34].

### 2.3. Inclusion/Exclusion Criteria

Patients aged 18 or older with at least one ECG recording were included. For the initial analysis, we used the ECGs recorded prior to, yet closest to, the FCHD event, with no restriction on the time between the ECG and FCHD event. These ECGs were identified through the recorded acquisition time of the ECG and the difference between this acquisition time and the time of the event. For controls, ECGs from the date last seen in the EHR system were used as the anchor point for right censoring.

### 2.4. Electrocardiogram Data

Raw 10 s digital supine 12-lead electrocardiogram data were obtained from the EPIPHANY Cardiology Information System at the UTHSC. The 12-lead ECG data were at either a 500 Hz or 250 Hz sampling rate. All the ECGs were downsampled to 250 Hz by simple linear interpolation. The ECGs from AHWFB were exported from the GE MUSE cardiology information system server. All the ECGs from AHWFB were at 500 Hz and were downsampled to 250 Hz using the same method as applied to the ECGs from UTHSC. No major differences were observed between the ECGs obtained from the two systems. In addition, we removed the first second of the 10 s ECGs to reduce typical (physical) noise associated with ECG initiation, such as patient movement. We also developed a single-lead version using ‘Lead I’ of the 12-lead ECG, since this lead is typically mimicked in wearable devices with single-lead ECG functionality such as smart watches and fitness trackers.

### 2.5. Risk Factors

We also included demographic and clinical risk factors obtained from the EHRs that were available to us. Demographic variables included age, sex and race and clinical risk factors included diabetes, hypertension, atrial fibrillation (AF), valvular disease (VD), coronary artery disease (CAD) and left ventricular hypertrophy (LVH). In addition to the risk factors included in the model development, we also assessed the statistical differences between the cases and controls (for FCHD) using additional co-morbidities that are related to a high risk of FCHD from the AHWFB patient population. Such clinical data include cardiomyopathy, dilated cardiomyopathy, obstructive hypertrophic cardiomyopathy (HCM), cardiomyopathy with reduced ejection fraction (EF), arrhythmia and amyloidosis.

### 2.6. Study Design, Model Development and External Validation

The UTHSC data were split in a stratified way into 80% for derivation and 20% as a holdout dataset. The 20% holdout set was kept completely independent for final testing and performance reporting. Five-fold cross-validation (80–20% split) was employed on the 80% derivation data, resulting in five different models being developed, where each model is tested on 20% of the derivation dataset. After cross-validation and the obtaining of the best evaluation metrics, the final models were tested on the holdout dataset.

The UTHSC data split (80% training, cross-validation folds and 20% holdout) was kept identical for all the developed models in this study. Each model was assessed using the area under the receiver operating characteristics curve (ROC AUC) for all the models and a concordance index (C-Index) for the Cox proportional hazard regression models. The model performance was assessed based on the results of the UTHSC 20% holdout data. Within the holdout dataset, only one ECG (latest ECG) per patient was included. To ensure the reliability of the model development, we split the data into training, validation and holdout at patient level, preventing any data leak between training, validation and holdout test. We then externally validated the FCHD prediction model AHWFB data.

All model development and analyses were performed using the Python programming language.

### 2.7. Prediction of Fatal Coronary Heart Disease Using Raw ECGs

This research employed an adjusted ResNet convolutional neural network (CNN) deep learning architecture, outlined in He et al. [35] and modified by Akbilgic et al. for ECGs [25], to predict the risk of FCHD. Dropout layers were included in the architecture, and regularization values were tuned to reduce the potential of the ECG-AI model to overfit the data. For training, the batch size (the number of data points evaluated at a time to update the model hyperparameters) was set to 1024 and training occurred over 100 epochs. The Adam optimizer was used as the optimization algorithm for the ECG-AI deep learning network.

The input into this deep learning architecture is a one dimensional 12-lead ECG signal and the output was the predicted risk of FCHD. Effectively, the one-dimensional input is an array that constitutes 2250 rows (since the sampling rate was downsampled to 250 Hz, resulting in 9 s at 225 Hz) of amplitudes and 12 columns, one for each of the 12 leads of the ECG. For the single-lead version, the architecture was kept the same, where Lead I was replicated twelve times to ensure homogeneity between the methods and architecture used.

All the model development and associated analyses were performed using the Python programming language (Python V3.10) and TensorFlow (V2), in combination with scikit-learn, were used to develop the ECG-AI model. The scikit survival package was the main package used to develop the Cox proportional hazard models and perform the associated analyses.

### 2.8. Survival Analysis with Cox Proportional Hazard Models

Multiple Cox proportional hazard models were developed using (i) demographic data, (ii) demographics plus clinical data and (iii) an additional four models that incorporate the 12- and single-lead ECG-AI output with demographic and clinical data (ECG-AI-Cox models). The ECG-AI-Cox models were assessed using the concordance index (C-Index) and time-dependent AUC. Time-dependent AUC is the AUC at each time point from the time the ECG was taken until the incident [36].

### 2.9. Subgroup Analyses

Subgroup analyses were performed using the UTHSC holdout and AHWFB data, for six different age groups, sex, race, diabetes, hypertension valvular disease and atrial fibrillation as risk factors to FCHD. The statistical significance of the difference between AUCs for the subgroups was assessed using DeLong’s test.

### 2.10. 12- and Single-Lead ECG-AI Correlation

Correlation between the predictions from the 12- and single-lead ECG-AI models, for both the UTHSC and AHWFB data, was assessed using the coefficient of determination, Pearson Correlation, Spearman Correlation coefficients and Cohen’s Kappa. The AUCs were statistically compared using DeLong’s test. In addition, we performed risk stratification of the 12- and single-lead ECG-AI models to compare risk predictions.

## 3. Results

### 3.1. Clinical Characteristics of the UTHSC and AHWFB Cohorts

The analytical (UTHSC) cohort (Table 1) included a total of 167,662 ECGs collected from a total of 50,132 patients after applying inclusion and exclusion criteria. Of these, 29,093 were controls with 78,472 ECGs and 21,039 were cases of FCHD with 89,190 ECGs. In some situations, the same visit had multiple ECGs and, therefore, all available ECGs were used. The patient cohort had an average age (age at time the ECG was taken) of 62.58 ± 14.0 (61.63 ± 13.81 for controls and 63.90 ± 14.10 for cases), of which 51.69% were African American (45.50% of controls and 60.24% of cases) and 45.72% were White (51.79% of controls and 37.31% of cases). Within the entire cohort, 53.09% were males (54.04% of controls and 51.78% of cases). The mean time between the ECG and FCHD was 2.25 ± 2.68 years (median = 1.38; minimum = 0, maximum = 24 years).

The external validation cohort from the AHWFB cohort had 10,406 ECGs from 2365 patients (3649 ECGs from 464 cases and 6757 ECGs from 1901 controls). From AHWFB, one ECG taken closest to the event per participant was used. After exclusion, a total of 2305 patients were included for external validation with 461 cases for FCHD. The median time between the ECG and FCHD was 1.06 years. The patient cohort had an average age (SD) of 62.58 (14.0), 61.71 (17.06) for controls and 68.40 (15.02) for cases, of which 18.79% were African American (17.41% of controls and 24.30% of cases) and 75.75% were White (76.31% of controls and 73.54% of cases). Within the entire cohort, 51.02% were males (48.92% of controls and 59.44% of cases). In addition, we also report some additional differences within the AHWFB patient population related to high-risk factors (Table S1). In brief, the FCHD cases had a high rate of arrhythmia (41.7%) with a high statistical significance (*p* < 0.0001) when compared to the controls. Similarly, cardiomyopathy is also comparatively high between cases and controls (9.8% cases vs. 6.0% controls), increasing the potential risk of FCHD. The additional risk factors assessed were at relatively low event rates in both cases and controls.

### 3.2. FCHD Risk Prediction Model Evaluations for the UTHSC Testing Cohort and AHWFB Validation Cohort

The 12- and single-lead ECG-AI models resulted in an AUC of 0.77 (0.76–0.78) and 0.76 (0.76–0.77), respectively, on the 20% University of Tennessee Health Science Center (UTHSC) holdout dataset (i.e., final testing dataset) and externally validated on the Atrium Health Wake Forest Baptist (AHWFB) dataset (only used for external validation) with AUCs of 0.84 (0.81–0.86) and 0.85 (0.83–0.87), respectively. Based on the UTHSC holdout data, the highest AUC of 0.85 (0.84–0.86) and C-Index of 0.78 (0.77–0.79) were achieved when the 12-lead ECG-AI predictions were combined with the demographic and clinical variables (C12-ECG-AI-Cox model). Based on the AHWFB data, the highest results were obtained for the D1-ECG-AI-Cox (C-Index = 0.83 [0.80–0.85], AUC = 0.87 [0.85–0.89]), which incorporates demographic data with single-lead ECG-AI predictions, and C1-ECG-AI-Cox models, which incorporate demographics + clinical data + single-lead ECG-AI predictions with the same C-Index and AUC (See Table 2 for all alternative model comparisons).

We also developed models on just demographic data (D-Cox) and demographic plus clinical data (C-Cox). In the UTHSC and AHWFB cohorts, the D-Cox model (demographic data only) resulted in a C-Index of 0.60 (0.59–0.61) and AUC of 0.65 (0.63–0.67) based on the UTHSC holdout data and a C-Index of 0.63 (0.61–0.67) and AUC of 0.68 (0.65–0.71) based on the AHWFB data. The C-Cox (demographics plus clinical data) model resulted in a C-Index of 0.66 (0.65–0.67) and AUC 0.69 (0.68–0.70) based on the UTHSC holdout dataset and a C-Index of 0.65 (0.62–0.68) and AUC of 0.69 (0.67–0.72) based on the AHWFB external validation cohort.

Using the AHWFB validation data, D1-ECG-AI-Cox and C1-ECG-AI-Cox were the best performing models. The D1-ECG-AI-Cox model was chosen as the best overall model due to the significant improvement in AUC based on the externally validated cohort data as well as the simplicity of its inputs (age, race, sex and ECG-AI prediction). This model had an accuracy of 83%, sensitivity of 69% and specificity of 86% based on the AHWFB dataset. Coefficients for all Cox models are provided in Table S2 and a summary of all evaluation metrics for each model tested on both cohorts are provided in Supplementary Material Table S3. To compare the D1-ECG-AI-Cox model with a ‘model’ that would often be used in clinical settings, i.e., using demographics and clinical data without ECG information (C-Cox), we also performed risk stratification and concordance between results from these two models (Table 3). This resulted in a kappa of 0.23, Spearman’s correlation of 0.54 and Pearson’s correlation of 0.46. Both models agreed in placing patients in the low-risk group 72% of the time while agreement decreased to 53% for high-risk classification. There was a total of 66% overall agreement between the D1-ECG-AI-Cox and the C-Cox models in FCHD risk classification.

The DeLong test for significant differences in AUCs between the two cohorts (Table 2) show that there was a significant difference between the D1-ECG-AI-Cox AUCs (*p*-value < 0.001) and C1-ECG-AI-Cox AUCs (*p*-value = 0.011) but no significant difference (*p* = 0.744) between the C12-ECG-AI-Cox AUCs. There was also a significant difference (*p* < 0.05) when the 12- and 1-lead ECG-AI model performance were compared between the UTHSC and AHWFB data, with AUCs (AUC = 0.84 and 0.85 for 12-ECG-AI and 1-ECG-AI, respectively) significantly higher based on the latter dataset.

### 3.3. Correlation and Concordance Analysis with Risk Stratification Using ECG-AI

When comparing the 12-lead based and single-lead-based ECG-AI models, i.e., ECG-only models based on the UTHSC cohort and the AHWFB validation cohort, separately, there was no statistical significance between the two AUCs (DeLong *p*-values of 0.153 for ECG-AI models on UTHSC data and 0.550 on AHWFB data).

#### 3.3.1. Correlation and Stratification Between 12-Lead and Single-Lead ECG-AI Predictions

In this subsection, we assessed the correlation and concordance between the exact numerical predictions obtained from 12-lead and single-lead models. The predictions based on the UTHSC cohort results are highly correlated between the two, with a Pearson Correlation Coefficient of 0.85 and Spearman Correlation Coefficient of 0.85 and concordant with Cohen’s Kappa of 0.68. The predictions based on the AHWFB validation cohort show moderate to strong correlation and concordance, resulting in Pearson Correlation Coefficient of 0.74, Spearman Correlation Coefficient of 0.68 and a comparable Cohen’s Kappa of 0.64.

#### 3.3.2. Comparison of Risk Stratification for 12- and Single-Lead ECG-AI Predictions

In this subsection, we assess the concordance between the binary decision (low or high risk) made by the 12-lead and single-lead ECG-AI models. When stratifying the patients by FCHD risk within the AHWFB cohort (Table 4), 86% of the time both ECG-AI models produced the same class prediction. In total, 169 out 1687 of patients (10%) predicted as low risk by the single-lead ECG-AI were predicted as high risk by the 12-lead ECG-AI model, while 157 out of 618 patients (25%) predicted as high risk by the single-lead ECG-AI were predicted as low risk by the 12-lead ECG-AI.

For comparison, we also stratified patients at low or high risk for FCHD using both ECG-AI models for the UTHSC holdout cohort. Overall, for this cohort, 84% of the time, both ECG-AI models produced similar class predictions. In further detail, 842 of 5781 people (15%) who were predicted as low risk by the single-lead-based ECG-AI were predicted as high risk by the 12-lead-based ECG-AI while 717 of 4237 patients (17%) predicted as high risk by the single-lead based ECG-AI were predicted as low risk by the 12-lead-based ECG-AI (Table S4).

### 3.4. Time-Dependent Analysis and Short-Term FCHD Risk Predictions

The 2-year FCHD event rate was 24% in the UTHSC and 18% in the AHWFB datasets. Time-dependent analysis was performed using the model chosen as the best overall, the D1-ECG-AI-Cox model (demographics and single-lead ECGs as inputs), resulting in an AUC of 0.91 (0.89–93) for 2-year FCHD risk prediction on the AHWFB cohort (Table 5), reducing to 0.88 (0.86–0.90) for 5-year risk prediction. The accuracy of this model is 82% with a specificity of 83%, sensitivity of 74%, PPV of 53% and NPV of 93% (Table 5). For most models, the predicted risk of FCHD after 5 years only slightly reduced in accuracy, with the AUC stabilizing until the 10-year risk prediction but often further reduced in accuracy after the 10-year mark. For example, the AUC for the best operating model (D1-ECG-AI-Cox) reduced to ~0.74.

Since the two-year FCHD prediction resulted in a high AUC when using the D1-ECG-AI-Cox model, which only includes demographic data and the ECG-AI prediction, we also assessed the ECG-only models (i.e., without the demographic data) for their power to predict FCHD within 2 years for comparison with the D1-ECG-AI-Cox model. For this, FCHD events within two years were labeled as ‘1’ and any event after two years were labeled as ‘0’ to identify the controls who did not have a FCHD event within this period. On the UTHSC holdout data, both the 12- and 1-ECG-AI models increased in AUC from 0.77 to 0.80 (0.79–0.81) and AUC of 0.76 to 0.78 (0.77–0.80), respectively. On the external validation cohort (AHWFB), the 12-ECG-AI resulted in an AUC of 0.84 (0.81–0.6) and the 1-ECG-AI resulted in an AUC of 0.86 (0.84–0.88), showing a slight improvement in accuracy for the latter model.

### 3.5. Subgroup Analyses

We performed subgroup analysis on age groups, sex and race and co-morbidities using the best performing D1-ECG-AI-Cox model based on the AHWFB dataset (Table 6 and Table S6) and on the UTHSC holdout dataset (Tables S5 and S6) for comparison, acknowledging that accuracy was lower when tested on the latter. In the AHWFB cohort, subgroup analysis showed no significant difference when comparing males and females, with AUCs of 0.85 (0.83–0.88) and 0.83 (0.79–0.86) and *p* = 0.286. There was also no significant difference (*p* = 0.524) in model performance between African American vs. White people, with AUCs of 0.86 (0.81–0.90) and 0.84 (0.82–0.87), respectively. The same non-significance was observed in the same subgroups within the UTHSC holdout cohort, albeit with lower accuracy (AUC 0.73 for females, 0.76 for males and 0.74 for both races. Within the AHWFB cohort, there were also no significant differences in AUCs for subgroups when tested on associated risk factors. However, when tested on the UTHSC holdout data, there were significant differences in the AUC (*p* < 0.001) for patients with/without hypertension and diabetes.

The subgroup results (Table 6 and Table S7) for the different age groups in the AHWFB validation cohort showed that the highest AUC of 0.96 was achieved for the age groups 18–29, with AUC = 0.87 for 60–69-year-olds. There were no significant differences between most age groups except when comparing the AUCs for the 18–29-year-olds with the other age groups (*p* < 0.05). However, we would like to note that the AHWFB cohort had a very small number of patients under the age of 29. This significant difference was not observed when the same analysis was performed using the UTHSC holdout data. Similarly, there were no significant differences between most age groups within the UTHSC cohort, especially for age ranges between 18–69. However, there were significant differences when comparing the 70+ age groups with most of the other age groups (*p* < 0.05; Table S6).

## 4. Discussion

In this research, we developed and compared multiple models to assess the power of ECGs to predict the risk of FCHD and assess predictive accuracy over time. The results show that the highest AUC (AUC = 0.87) was obtained when using age, sex, race and single-lead (Lead I) ECGs. In addition, adding clinical risk factors provided the same accuracy, yet we determined that demographics plus Lead I ECGs was the best model since its execution relies on fewer and easier to obtain data points. Time-dependent analysis using this best model resulted in an AUC of 0.91 in predicting the 2-year risk for FCHD. In addition, based on the AHWFB external validation cohort, the model using only Lead I ECGs (with no additional data) resulted in an AUC of 0.86 (0.84–0.88) when tested on the 2-year FCHD prediction. Despite the accuracy being lower than the AUC of 0.91, this shows that a Lead I ECG on its own, without the need for any additional information about the patients, can result in a very high accuracy in determining FCHD risk. Our conclusion is also supported by the results of this research, showing that there is no significant difference in FCHD risk prediction when using only single-lead ECGs instead of all 12 leads. These results may be seen as a milestone for future studies aiming to develop FCHD risk prediction remotely using consumer level smart watches with ECG functionality.

The major problem with FCHD is the suddenness and rapidity with which the event is fatal. While there are multiple risk factors associated with FCHD, it is often difficult to ascertain which risk factors are the highest contributors to the event. In this study, using data available to us from the AHWFB cohort, we assessed whether additional co-morbidities not included within the model development were significantly different between the cases and controls. Our results show that arrhythmia and cardiomyopathy (Table S1) can indeed be contributors to an increased FCHD event rate. This information, however, can be very limited in the patient population that could be at high risk of suffering from an FCHD event. Therefore, there is a need for prediction models that can be easily accessible and used within routine clinical care to increase monitoring and follow-ups. While some time-dependent FCHD analyses have been previously performed, high accuracy was achieved up to 24 h before sudden arrest and moderate to low accuracy up to 10 years before the event [5]. Kwon et al. [23] demonstrated that in-hospital sudden cardiac arrest could be predicted in >50% of patients several hours before the event using machine learning. However, predicting SCD in such a short time period may not be clinically helpful for intervention. Therefore, using simple and routinely collected data, e.g., ECGs, can be an asset within AI models to increase pre-screening efforts for fatal events. In a large-scale prospective study, it was demonstrated that deep learning artificial intelligence could successfully predict cardiac arrest using diverse formats of ECGs, mostly depending on ECG-based features and data for the amplitude and duration of individual ECG waves [23]. However, the use of numerous ECG features can be difficult to obtain and might not always be available from clinical institutes, increasing the need for ECG-AI methods using raw ECGs in predicting outcomes.

In some published AI-driven FCHD or SCD prediction research, very high accuracies (AUC > 0.85) were achieved hours before the event, which reduces their usability for therapeutic interventions as well as not being applicable to population-level screening [5,18,37]. The results of our research show a sustained high AUC of 0.91 for the 2-year risk prediction of FCHD using only age, sex, race and Lead I ECG data. In addition, we also show that ECG-based predictions substantially improve model predictions when compared to models developed using only demographics or demographics plus clinical data. This highlights the importance of ECG-based models and assessments years before the potential FCHD incident. In this period, the model can distinguish between FCHD and non-FCHD events (NPV = 93%, PPV = 53%), making it increasingly useful if incorporated within clinical settings as a tool for risk assessment. It is interesting to note that, overall, accuracy was higher for most of the ECG-AI-based models when tested on the external validation set (AHWFB). This could be related to different patient profiles between the two clinical settings and could also relate to socio-demographic features that were not included as influencing factors within the models. In addition, there could also be some signal resolution differences between the EPIPHANY and GE MUSE systems which might not have been fully accounted for. Also, it is possible that the accuracy of the ICD coding may be different from one institute to another, resulting in different validation accuracies.

Furthermore, following the work in this research on the feasibility of a single-lead ECG-AI model, the results show that the 12- and single-lead ECG-based predictions are strongly correlated. When risk stratification was performed, the overall agreement between the two cohorts was strong and similar (84% agreement vs. 86% for UTHSC and AHWFB, respectively). In addition, in this work, validation based on the external cohort resulted in higher accuracy of the ECG-AI plus demographic data, with similar AUCs when the single-lead ECG-AI (1-ECG-AI) was also used. We would like to note that the similarity in AUC between the ECG-only models and the ECG + demographic (D1- and D12-ECG-AI) models can indicate that ECG-AI on its own can be a powerful tool. For further simplicity and ease of deployment, while retaining high accuracy, using only ECG data without the need for collection of demographic data can be a substantial asset, acknowledging there is a slight trade-off between accuracy and simplicity.

Nevertheless, the higher accuracy in the single-lead-based models paves the way for integration within mobile AI platforms such as ECG-Air [38], which can gather ECG data from wearable devices such as smart watches for easier, simpler and cost-effective pre-screening at the population level. While 12-lead ECGs are the standard in clinical practice, there is often more background ‘noise’ and deviations in the waveform, and they are more prone to lead misplacement, which can be attributed to sub-optimal environments and patient movements when the ECG is being performed [39,40]. However, Lead I of a 12-lead ECG often has less ‘noise’ or artifacts [39].

We would like to note that the patient population from both UTHSC and AHWFB had an average age of over 60. While younger patients were included in the cohorts, these were at relatively smaller percentages. Therefore, further testing of the developed models is required on a younger cohort. However, we would also like to point out that, following subgroup analyses, acknowledging the differences in percentages between age groups, the results show that the best operating model (D1-ECG-AI-Cox) can perform well in most age groups (Table 6).

We acknowledge that the use of a 12-lead ECG in combination with demographic/clinical data in an incorporated AI data-driven model within the clinical workflow may raise questions about its cost-effectiveness and feasibility issues. First of all, the ECG-AI models utilize waveform ECG data, and such waveform data are almost never transferred to EHRs. They are typically encoded or encrypted and kept on different servers, often referred to as cardiology information servers. The deployment of ECG-AI models in the clinical workflow would require infrastructural investments that can pull waveform data from cardiology information systems, run the ECG-AI models and return the outcomes into EHRs. While this is perfectly achievable, it requires technical expertise and ECG system vendors willing to cooperate. It also depends heavily on less accessible and costly medical imaging modalities such as cardiac MRI. Despite this, such AI models may result in significantly increased risk prediction and disease detection accuracies, but only people who have access to such imaging modalities can benefit from them, which would deepen health disparities. However, ECGs are comparatively low cost and more accessible compared to other cardiac imaging modalities. Therefore, the deployment of ECG-AI models may result in a minimal number of added disparities or, potentially, reduced healthcare disparities by enabling early diagnosis of major conditions only from ECGs, which otherwise would require more expensive data modalities.

At this point, it is very important for hospital systems interested in such ECG-AI models to ensure that their ECG vendors are kept obligated to make ECG waveform data available in a non-encrypted fashion. Indeed, there is a need to enforce such data availability requirement at the national level for any EGG vendor doing business in that country. In terms of cost effectiveness, we acknowledge that there is an infrastructure cost needed to deploy such models into the clinical workflow. However, this cost would likely be less than the cost of ICD placement in 5 to 10 patients or the cost of annual care of 5 to 10 heart failure patients. Also accounting for potentially improved patient outcomes, the benefits of having ECG-AI deployment technologies can be superior to the associated clinical costs.

While these challenges will remain, some, such as cost-effectiveness and clinical burden, can be reduced by the potential to use the single-lead ECG-AI (with demographics) model in remote devices, e.g., smart watches. Such smart devices with ECG-capturing technologies have made the use of Lead I in AI models more accessible, increasing the usability of single-lead ECG-AI models in the near future. In addition, more and more people have access to health monitoring applications on their smart watches, linked to their phones. Therefore, having a direct AI-based ECG model that can provide information on cardiovascular health, without alarm fatigue, can be a very important aspect in increasing awareness and reducing the rates of fatal diseases, such as FCHD.

The availability of single-lead AI based tools for pre-screening are becoming more important since FCHD and related sudden arrest have shown increased prevalence in the younger generation and in athletes as well as in people with certain cardiac diseases such as HCM, the larger subset of people with LVH and some risk factors, e.g., diabetes [41,42,43]. In the model using clinical data and 12-lead (and single-lead) ECGs, LVH and diabetes all showed high coefficients in the respective ECG-AI-Cox models (Table S3). For all the ECG-AI based Cox models, the ECG-AI prediction was always consistently higher in its predictive importance.

In conclusion, having a simple and usable AI model that can be integrated within a smart device, especially a smart watch with single-lead ECG capabilities, can be an asset to the clinical workflow and help decision making for triaging, testing and risk assessment. The high accuracy of using Lead I of a 12-lead clinical ECG suggests that, after further work, single-lead ECGs from smart watches may be used to monitor larger patient populations for FCHD.

### Limitations, Translational Outlook and Future Studies

Our results are EHR-based and thus represent real-world scenarios. Yet, in the EHR settings, almost all ECGs are recorded due to a clinical condition, suspicion of a medical condition or follow-up for such conditions. Therefore, the use of our ECG-AI models for the screening of large patient populations requires further study within the general population, where the ECGs are not recorded as part of routine care. To address these limitations, our current efforts are focusing on gathering a larger set of data from established NHLBI-funded cohort studies for additional validation.

Despite our models showing very high accuracy to identify patients at risk of FCHD, there is high enough accuracy to prove its clinical utility, even if it may not alter the event, but can help with identifying people at high risk. An additional limitation is the potential differences in signal resolution between the two ECG systems (i.e., EPIPHANY and GE MUSE). While in this work, raw ECGs were used with minimal adjustments, this could have created a limitation and reduced accuracy within the UTHSC holdout set. Further testing is required to understand changes in signal resolution and whether such changes impact model accuracy.

In this study, the event rate of FCHD in our study is high and this potentially stems from two reasons. First, we consider up to 24 years for incidents of FCHD. Second, our cohort was limited to patients who were admitted to hospital for a medical reason as part of their standard of care, which could have skewed the cohort towards relatively higher risk patients. To address these limitations, there are two main priorities for future studies. Primarily, the developed models, both the 12-and 1-lead ECG-AI models, as well as the associated Cox models, would benefit from blinded validation from an additional institute, where the patient profiles (including age, race and event rates of co-morbidities) are different to those from UTHSC and AHWF. In addition, future work should include another validation based on cohort studies where ECGs were recorded as part of study protocol rather than on the basis of medical need. Furthermore, this study utilizes raw ECG waveforms rather than ECG characteristics, adding some limitation to identify the characteristics of the ECG signals that are indicating a risk of FCHD. Our planned future work will include the use of explainable AI to highlight and extract characteristics of ECGs that could potentially show FCHD risk.

In addition, future work will also focus on AI-assisted clinical trials to identify high-risk patients, randomize them into standard of care and intervention, and observe the differences in outcome. FCHD risk is difficult to fully ascertain, especially from the EHR system and, therefore, there might be a risk of misidentification or under-reporting of patients who had FCHD. Although this study assessed the correlation of 12-lead and single-lead models, feasibility studies for future remote monitoring via wearables (e.g., smart watches) is still ongoing [31] and require a larger cohort for prospective studies.

## 5. Conclusions

The results of this research indicate that FCHD events can be predicted from both Lead I ECGs and from Lead I ECGs in combination with age, gender and sex with very high accuracy. The strong correlation of Lead I ECG-based results with 12-lead ECG models, and also with ECG plus clinical data-based models, suggest that the information from the other 11 ECG leads and well as clinical risk factors are inherently encoded into Lead I of an ECG explaining why Lead I ECG-alone models can yield a high FCHD risk prediction accuracy. The high accuracies obtained in external validation and our subgroup analysis suggest the generalizability of our models. If their clinical utility is proven, such models can be embedded into telehealth and mobile health systems to monitor larger patient populations at low cost and become accessible to under-represented groups and minorities.

## 6. Clinical Perspectives

- FCHD is a major public health concern and is a sudden event; therefore, it requires early prediction and detection to ensure appropriate risk management and therapeutic intervention.
- ECG-AI models and models incorporating ECG-AI with demographic data have been validated with high accuracy, increasing their potential to be incorporated within clinical workflows.
- High 2-year FCHD prediction accuracy can guide developing novel protocols for detailed examination and close monitoring of those identified at high risk.
- The strong correlation between 12-lead and single-lead ECG-AI predictions showcases the usability of simple and cost-effective screening tools, which can help clinical decision making by using smart watches available on the market.
- Incorporating the use of such AI-based models has the potential to help with clinical assessment and develop patient-centered risk management schemes.

## Figures and Tables

**Table 1:** Demographics and clinical characteristics of the patient cohorts of UTHSC and AHWFB.

	UTHSC				AHWFB	
Demographics/Risk Factors	Total	Controls	Cases	Total	Controls	Cases
	N_patients_ = 50,132	N_patients_ = 29,093	N_patients_ = 21,039	N_patients_ = 2305	N_patients_ = 1844	N_patients_ = 461
Age at ECG taken (years) ± SD	62.58 ± 13.98	61.63 ± 13.81	63.90 ± 14.10	63.04 ± 16.89	61.71 ± 17.06	68.40 ± 15.02
18–29	693 (1.38)	487 (1.67)	206 (0.98)	103 (4.47)	94 (5.10)	9 (1.95)
30–39	2069 (4.13)	1253 (4.31)	816 (3.88)	143 (6.20)	129 (7.00)	14 (3.04)
40–49	5809 (11.59)	3607 (12.40)	2202 (10.47)	220 (9.54)	198 (10.74)	22 (4.77)
50–59	11,822 (23.58)	7147 (25.57)	4675 (22.22)	401 (17.40)	332 (18.00)	69 (14.97)
60–69	13,970 (27.87)	8293 (28.51)	5677 (26.98)	540 (23.43)	432 (23.43)	108 (23.43)
≥70	15,769 (31.45)	8306 (28.55)	7463 (35.47)	898 (38.96)	659 (35.74)	239 (51.84)
Sex						
Male (0), n (%)	26,619 (53.10)	15,723 (54.04)	10,896 (51.79)	1176 (51.02)	902 (48.92)	274 (59.44)
Female (1), n (%)	23,513 (46.90)	13,370 (45.96)	10,143 (48.21)	1129 (48.98)	942 (51.08)	187 (40.56)
Race/Ethnicity						
African American, n (%)	25,911 (51.69)	13,238 (45.50)	12,673 (60.24)	433 (18.79)	321 (17.41)	112 (24.30)
White, n (%)	22,918 (45.72)	15,067 (51.79)	7851 (37.31)	1746 (75.75)	1407 (76.31)	339 (73.54)
Other or Mixed Race, n (%)	1303 (2.60)	788 (2.71)	515 (2.45)	126 (5.5)	116 (6.29)	10 (2.17)
Diabetes mellitus						
Yes (1), n (%)	20,290 (40.47)	8337 (28.67)	11,953 (56.81)	677 (29.37)	485 (26.30)	192 (41.65)
Hypertension						
Yes (1), n (%)	42,630 (85.04)	24,855 (85.43)	17,775 (84.49)	1395 (60.52)	1070 (58.03)	325 (70.50)
Left ventricular hypertrophy						
Yes (1), n (%)	243 (0.48)	0 (0.00)	243 (1.15)	37 (1.61)	23 (1.25)	14 (3.04)
Coronary Artery Disease						
Yes (1), n (%)	42,261 (84.30)	24,000 (82.49)	18,261 (86.80)	1958 (84.95)	1601 (86.82)	357 (77.44)
Valvular disease						
Yes (1), n (%)	1298 (2.59)	383 (1.32)	915 (4.35)	369 (16.01)	267 (14.48)	102 (22.13)
Atrial Fibrillation						
Yes (1), n (%)	12,562 (25.06)	4347 (14.94)	8215 (39.05)	204 (8.85)	134 (7.27)	70 (15.18)

**Table 2:** C-index and/or AUC results of the developed models, evaluated on the 20% holdout set and validated on the AHWFB dataset. In **bold** is the best overall model chosen for accuracy and simplicity.

	UTHSC 20% Holdout		AHWFB		
Model Name	C-Index (95% CI)	AUC (95% CI)	C-Index (95% CI)	AUC (95% CI)	DeLong Test (*p*-Value)
** 12-ECG-AI		0.77 (0.76–0.79)		0.84 (0.81–0.86)	<0.001
** 1-ECG-AI		0.76 (0.75–0.77)		0.85 (0.83–0.87)	<0.001
^†^ D-Cox	0.60 (0.59–0.61)	0.65 (0.63–0.66)	0.63 (0.61–0.67)	0.68 (0.65–0.71)	0.058
^†^ C-Cox	0.66 (0.65–0.67)	0.72 (0.71–0.73)	0.65 (0.62–0.68)	0.69 (0.67–0.72)	0.055
^^^ D12-ECG-AI-Cox	0.72 (0.71–0.73)	0.81 (0.80–0.82)	0.80 (0.78–0.83)	0.85 (0.82–0.87)	0.001
^^^ **D1-ECG-AI-Cox**	**0.70 (0.69–0.71)**	**0.79 (0.78–0.80)**	**0.83 (0.80–0.85)**	**0.87 (0.85–0.89)**	**<0.001**
^^^ C12-ECG-AI-Cox	0.78 (0.77–0.79)	0.85 (0.84–0.86)	0.81 (0.78–0.83)	0.85 (0.83–0.87)	0.744
^^^ C1-ECG-AI-Cox	0.76 (0.75–0.77)	0.84 (0.83–0.86)	0.83 (0.80–0.85)	0.87 (0.84–0.89)	0.011

** *ECG-AI* Model (using only ECGs). No C-statistics are reported as these are classification models. ^†^ Cox proportional hazard models; ^^^ *ECG-AI-Cox* models (including demographics, clinical and ECG-AI predictions).

**Table 3:** Contingency table for risk stratification comparing D1-ECG-AI-Cox (chosen as best model) and C-Cox (model reflected in clinical settings) on the AHWFB external validation cohort.

		Demographics + Clinical + ECG-AI Predicted Group (D1-ECG-AI-Cox)		Total
		Low Risk	High Risk	
**Demographics + Clinical Predicted Group (C-Cox)**	**Low risk**	1186	313	1499
	**High Risk**	460	346	806
	**Total**	1646	659	2305

**Table 4:** Contingency table for risk stratification using 12-ECG-AI and 1-ECG-AI models on the AHWFB external validation cohort.

		1 Lead Predicted Group (1-ECG-AI)		Total
		Low Risk	High Risk	
**12 Lead Predicted Group (12-ECG-AI)**	**Low risk**	1518	157	1675
	**High Risk**	169	461	630
	**Total**	1687	618	2305

**Table 5:** Confusion matrix for the 2-year D1-ECG-AI-Cox prediction model on the AHWFB data.

Actual/Predicted	No FCHD	FCHD	
**No FCHD**	1539	305	Specificity = 83%
**FCHD**	121	340	Sensitivity = 74%
	Negative Predictive Value = 93%	Positive Predictive Value = 53%	Accuracy = 82%

**Table 6:** Subgroup analysis using the best operating model (D1-ECG-AI-Cox) on the AHWFB validation cohort. Comparisons were performed using the DeLong test. * Detailed subgroup analysis comparing all age groups is provided in Supplementary Material Table S7.

Subgroups	D1-ECG-AI-Cox AUC (95% CI)	DeLong Test *p*-Value
Sex		
Male	0.85 (0.83–0.88)	0.286
Female	0.83 (0.79–0.86)	
Age *		
18–29	0.96 (0.92–1.00)	Only significant difference is between 18–30 vs. all other age groups (see Table S7)
30–39	0.76 (0.59–0.92)	
40–49	0.81 (0.67–0.95)	
50–59	0.82 (0.76–0.87)	
60–69	0.87 (0.83–0.91)	
≥70	0.83 (0.80–0.86)	
Ethnicity		
African American	0.86 (0.81–0.90)	0.524
White	0.84 (0.82–0.87)	
Hypertension		
Yes	0.84 (0.81–0.86)	0.481
No	0.81 (0.73–0.89)	
Diabetes		
Yes	0.81 (0.78–0.85)	0.283
No	0.84 (0.81–0.88)	
Coronary Artery Disease (CAD)		
Yes	0.80 (0.75–0.85)	0.218
No	0.84 (0.81–0.87)	
Atrial Fibrillation (AF)		
Yes	0.79 (0.73–0.86)	0.275
No	0.83 (0.81–0.86)	
Valvular Disease (VD)		
Yes	0.79 (0.74–0.85)	0.090
No	0.84 (0.82–0.87)	

## Data Availability

The data used in this study is only available for IRB approved projects by Wake Forest University School of Medicine.

## Supplementary Materials

The following supporting information can be downloaded at: https://www.mdpi.com/article/10.3390/jcdd11120395/s1, Table S1: Event rate of additional co-morbidities that can increase risk of FCHD and assessment of statistical significance between FCHD cases and controls; Table S2: Coefficients of variables from the developed Cox/ECG-AI-Cox models; Table S3: Summary of evaluation metrics for each model tested on UTHSC 20% holdout data and AHWFB external validation; Table S4: Contingency table for risk stratification using 12-ECG-AI and 1-ECG-AI models on the UTHSC holdout data; Table S5: Subgroup analysis using the best operating model (D1-ECG-AI-Cox) on the UTHSC holdout cohort. Comparisons were performed using the DeLong test. * Detailed subgroup analysis comparing all age groups are provided in supplementary material Table S4; Table S6: DeLong test results (p-values) comparing the AUCs obtained for different age subgroups using the best performing model (D1-ECG-AI-Cox) on the UTHSC holdout cohort; Table S7: DeLong test results (p-values) comparing the AUCs obtained for different age subgroups using the best performing model (D1-ECG-AI-Cox) on the AHWFB cohort.
