# Supplementary material for: Time-Dependent ECG-AI Prediction of Fatal Coronary Heart Disease: A Retrospective Study
Source: J Cardiovasc Dev Dis. 2024 Dec 8;11(12):395. doi: 10.3390/jcdd11120395 (PMC11678222; doi:10.3390/jcdd11120395)
Supplement: Supplementary file 1 [file jcdd-11-00395-s001.zip › jcdd-3321844-supplementary.pdf]

Supplementary Material

Table S1 provides further information on the event rates of cardiac co-morbidities that could be related to a high FCHD event rate. Most notably, arrhythmia and cardiomyopathy had the highest (and significant) event rate in FCHD cases, and can correspond to high-risk patients.

Table S 1 Event rate of additional co-morbidities that can increase risk of FCHD and assessment of statistical significance between FCHD cases and controls

	All (n=2305)	Cases (n=461)	Controls (n=1844)	Chi square *p-value*
**Cardiomyopathy**	156 (6.8%)	45 (9.8%)	111 (6.0%)	<0.01
**Dilated cardiomyopathy**	47 (2.0%)	24 (5.2%)	23 (1.3%)	<0.0001
**Obstructive HCM**	13 (0.6%)	3 (0.7%)	10 (0.5%	0.078
**Cardiomyopathy with reduced EF**	19 (0.8%)	7 (1.5%)	12 (0.7%)	0.068
**arrhythmia**	572 (24.8%)	192 (41.7%)	380 (20.6%)	<0.0001
**amyloidosis**	12 (0.5%)	0 (0%)	12 (0.7%)	-

Table S2 provides the coefficients from the Cox models developed. In general, all the ECG-AI-Cox models resulted in the ECG-AI prediction to have the highest coefficient. Within the clinical ECG-AI models, diabetes, LVH followed the ECG-AI prediction with the next highest coefficients. In the C-Cox model, multiple related co-morbidities had comparatively high coefficients (CAD, diabetes, LVH and AF).

Table S2 Coefficients of variables from the developed Cox/ECG-AI-Cox models

Model Name	Sex	Race	Age at ECG	Valvular Disease	Atrial Fibrillation	Left Ventricular Hypertrophy	Coronary Artery Disease	Hypertension	Diabetes	ECG-AI Prediction
**D-Cox**	-0.13553	0.390791	0.022761							
**C-Cox**	-0.112565	0.323531	0.019638	0.122371	0.481184	0.634722	0.71379	-0.372812	0.6816	
**D12-ECG-AI-Cox**	-0.045086	0.111819	0.01683							2.660561
**C12-ECG-AI-Cox**	-0.044343	0.086839	0.016909	-0.010071	0.182525	0.411778	0.099484	-0.22867	0.508564	2.386135
**D1-ECG-AI-Cox**	-0.072165	0.156368	0.017776							2.733531
**C1-ECG-AI-Cox**	-0.068131	0.124767	0.017311	-0.004326	0.221302	0.437063	0.098059	-0.262053	0.536086	2.416201

Table S3 provides accuracy, specificity, sensitivity, negative predicted values (NPV) and positive predicted value (PPV) for all developed model developed. UTHSC refers to testing results on the 20% holdout dataset and AHWFB refers to the externally validated results.

Table S 3 Summary of evaluation metrics for each model tested on UTHSC 20% holdout data and AHWFB external validation

	Accuracy		Specificity		Sensitivity		NPV		PPV	
**Model Name**	**UTHSC**	**AHWFB**	**UTHSC**	**AHWFB**	**UTHSC**	**AHWFB**	**UTHSC**	**AHWFB**	**UTHSC**	**AHWFB**
**12-ECG-AI	70%	81%	71%	83%	68%	70%	75%	92%	63%	51%
**1-ECG-AI	70%	82%	71%	84%	67%	71%	81%	92%	55%	53%
†D-Cox	56%	62%	53%	62%	60%	60%	65%	86%	48%	28%
†C-Cox	67%	66%	72%	69%	61%	52%	72%	85%	61%	30%
^D12-ECG-AI-Cox	70%	84%	74%	89%	65%	62%	74%	90%	64%	59%
^D1-ECG-AI-Cox	69%	83%	72%	86%	64%	69%	74%	92%	62%	55%
^**C12-ECG-AI-Cox**	78%	83%	82%	88%	72%	62%	87%	90%	80%	57%
^C1-ECG-AI-Cox	72%	83%	75%	86%	68%	70%	77%	92%	66%	56%

Table S4 is a contingency table following risk stratification using the 12- and single-lead ECG-AI models on the UTHSC holdout data. Overall, both models predict similar risk and classify individuals similarly in both low and high risk groups.

Table S4 Contingency table for risk stratification using 12-ECG-AI and 1-ECG-AI models on the UTHSC holdout data

		**1 Lead Predicted Group (1-ECG-AI)**		
		**Low risk**	**High risk**	**Total**
**12 Lead Predicted Group (12-ECG-AI)**	**Low risk**	4939	717	5656
	**High Risk**	842	3520	4362
	**Total**	5781	4237	10018

Table S5 is a subgroup analysis performed using the D1-ECG-AI-Cox model on the UTHSC holdout cohort data. While this was not the model with the highest accuracy on the UTHSC holdout set, the D1-ECG-AI-Cox model was chosen as the best performing model on the AHWFB dataset but subgroup analyses were performed for comparison purposes.

Table S5 Subgroup analysis using the best operating model (D1-ECG-AI-Cox) on the UTHSC holdout cohort. Comparisons were performed using the DeLong test. *Detailed subgroup analysis comparing all age groups are provided in supplementary material Table S4.

**Subgroups**	**D1-ECG-AI-Cox AUC (95% CI)**	**DeLong Test p-value**
Sex		
Male	0.76 (0.74-0.77)	0.007
Female	0.73 (0.71-0.74)	
*Age**		
18-29	0.74 (0.64-0.84)	Only significant difference is between 70+ vs most other age groups (see Table S5)
30-39	0.78 (0.73-0.83)	
40-49	0.77 (0.74-0.80)	
50-59	0.76 (0.74-0.78)	
60-69	0.75 (0.73-0.77)	
≥70	0.71 (0.70-0.73)	
Race		
African American	0.74 (0.72-0.75)	0.851
White	0.74 (0.72-0.75)	
Hypertension		
Yes	0.73 (0.72-0.74)	<0.001
No	0.80 (0.78-0.82)	
Diabetes		
Yes	0.71 (0.70-0.73)	<0.001
No	0.75 (0.74-0.77)	
Coronary Artery Disease (CAD)		
Yes	0.75 (0.73-0.76)	0.531
No	0.74 (0.71-0.76)	
Atrial Fibrillation (AF)		
Yes	0.70 (0.68-0.72)	0.071
No	0.73 (0.71-0.74)	

Valvular Disease (VD)		
Yes	0.68 (0.60-0.75)	0.079
No	0.74 (0.73-0.75)	

Table S6 is a detailed subgroup analysis, on the UTHSC holdout data, comparing different age groups ranging from 18 to >70 years of age. Our models show no significant differences between most age groups, with significant differences resulting when comparing >70 year groups age groups above 30.

Table S6 DeLong test results (p-values) comparing the AUCs obtained for different age subgroups using the best performing model (D1-ECG-AI-Cox) on the UTHSC holdout cohort.

**Age Groups**	**18-29**	**30-39**	**40-49**	**50-59**	**60-69**	**≥70**
**18-29**						
**30-39**	0.483					
**40-49**	0.582	0.707				
**50-59**	0.718	0.417	0.545			
**60-69**	0.807	0.288	0.326	0.643		
**≥70**	0.629	0.010	0.001	<0.001	0.003	

Table S7 is a detailed subgroup analysis, on the AHWFB data, comparing different age groups ranging from 18 to >70 years of age. Our models show no significant differences between most age groups, with significant differences resulting when comparing 18-30 years groups with all other age groups.

Table S7 DeLong test results (p-values) comparing the AUCs obtained for different age subgroups using the best performing model (D1-ECG-AI-Cox) on the AHWFB cohort.

**Age Groups**	**18-29**	**30-39**	**40-49**	**50-59**	**60-69**	**≥70**
**18-29**						
**30-39**	0.020					
**40-49**	0.040	0.631				
**50-59**	<0.001	0.511	0.938			
**60-69**	0.002	0.199	0.421	0.136		
**≥70**	<0.001	0.411	0.805	0.719	0.111	
